# Organization and phylogenetic relationships of the mitochondrial genomes of *Speiredonia retorta* and other lepidopteran insects

**DOI:** 10.1038/s41598-021-82561-1

**Published:** 2021-02-03

**Authors:** Yu Sun, Hua Huang, Yudong Liu, Shanshan Liu, Jun Xia, Kai Zhang, Jian Geng

**Affiliations:** 1 Department of Health Inspection and Quarantine, School of Laboratory Medicine, Bengbu Medical College, Bengbu, 233004 China; 2 Research Center of Clinical Laboratory Science, School of Laboratory Medicine, Bengbu Medical College, 2600 Donghai Road, Bengbu, 233004 China; 3 Department of Stomatology, The First Affiliated Hospital of Bengbu Medical College, Bengbu Medical College, Bengbu, 233004 China

**Keywords:** Biochemistry, Biotechnology, Developmental biology, Evolution, Genetics, Zoology

## Abstract

In this study, we analyzed the complete mitochondrial genome (mitogenome) of *Speiredonia retorta,* which is a pest and a member of the Lepidoptera order. In total, the *S. retorta* mitogenome was found to contain 15,652 base pairs encoding 13 protein-coding genes (PCGs), 22 tRNAs, 2 rRNAs, as well as an adenine (A) + thymine (T)-rich region. These findings were consistent with the mitogenome composition of other lepidopterans, as we identified all 13 PCGs beginning at ATN codons. We also found that 11 PCGs terminated with canonical stop codons, whereas *cox2* and *nad4* exhibited incomplete termination codons. By analyzing the mitogenome of *S. retorta* using Bayesian inference (BI) and maximum likelihood (ML) models, we were able to further confirm that this species is a member of the Erebidae family.

## Introduction

*Speiredonia retorta* (Lepidoptera: Erebidae) is a pest species that is widely distributed throughout Southeast Asia. *S. retorta* larvae can adhere to *Acacia* leaves and branches where they undergo pupation, while adults can feed on a range of fruits including apples, pears, and grapes, leading to their more rapid decay. These moths produce three generations per year, with a life cycle consisting of an egg stage (6–18 days), a larval stage with six instars (23–47 days), and a pupal stage (8–13 days). These insects overwinter in the pupal stage, which can last from 194–228 days^1^. The primary predators of this species include *Ophion luteus* (Hymenoptera: Ichneumonidae), *Brachymeria obscurata* (Hymenoptera: Chalalcididae)*,* and *Exorista sorbillans* (Diptera: Tachinidae). Given that these moths represent a significant economic threat, their management is a key agricultural concern and necessitates an in-depth understanding of their biology. Further exploration of the phylogeny and genetic characteristics of *S. retorta* has the potential to offer novel insights into how to appropriately combat the spread of these moths. Understanding and analyzing the mitochondrial genome (mitogenome) of this species, in particular, may facilitate key comparative phylogenetic and evolutionary studies that can support such preventative efforts^2,3^.

The mitogenome of metazoan species generally ranges from 14,000–19,000 bp in size, with few or no intergenic spacer regions^4^, encoding 13 protein-coding genes (PCGs), 22 tRNAs, and 2 rRNAs^5,6^. In addition to these more broadly conserved features, members of the Lepidoptera order generally exhibit a conserved adenine and thymine (A + T)-rich region within their mitogenome. The mitogenome represents an ideal tool for the analysis of phylogenetic relationships, given that it has a simple structure, is maternally inherited, rarely undergoes recombination, and is conserved over the course of evolution^7–9^. Modern advances in sequencing techniques have led to the publication of mitogenomes from many different insects and other species, thereby supporting a wide range of evolutionary analyses^6^.

By conducting a comprehensive analysis of the mitogenome of a given insect species, we have the opportunity to perform intricate phylogenetic or population genetics studies and to identify potentially novel genes that may serve as valuable targets in future research efforts. The Lepidoptera order is the second-largest Insecta order and is composed of > 155,000 species of moths and butterflies^10^. Noctuoidea is the largest Lepidopteran sub-family, with > 42,400 species^11^. Several characteristic mitogenomic markers associated with these phylogenetic classes of insects have been identified to date, enabling us to reliably explore the phylogenetic relationship between *S. retorta* and other species through mitogenomic analyses. Six Noctuidae families were proposed in a phylogenetic framework constructed by Zahiri et al., including the Euteliidae, Erebidae, Nolidae, Notodontidae, Oenosandridae, and Noctuidae families^12^. Given the clear value of such analyses, in the present study we sequenced the full *S. retorta* mitogenome in an effort to more fully explore the evolutionary relationship between this agriculturally important insect and other Noctuidae species.

## Results and discussion

### Mitogenome structure, organization, and composition

**Figure 1:**
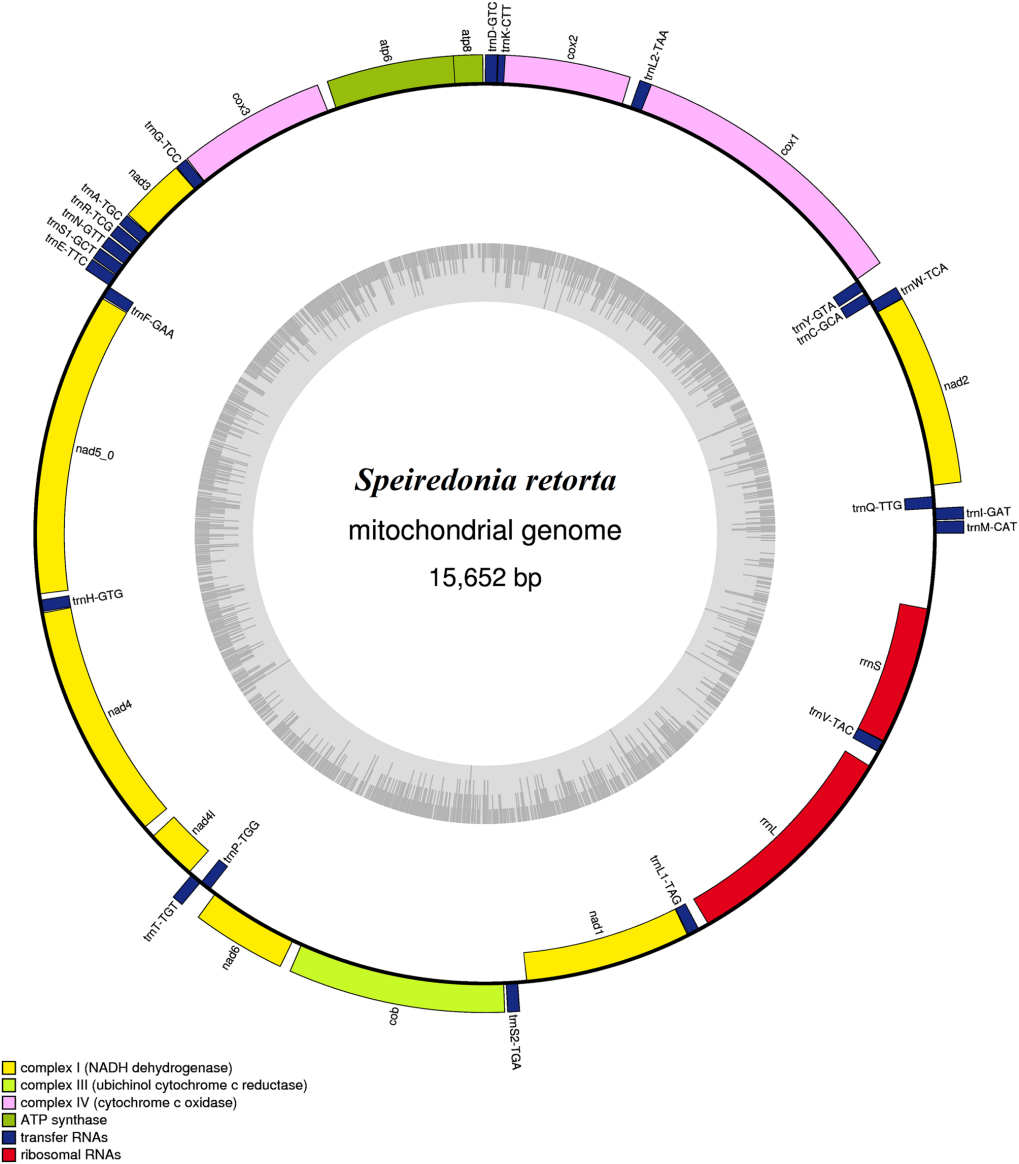
A map of the *S. retorta* mitogenome. Labeling of tRNA genes was conducted in accordance with IUPAC-IUB single-letter amino acid codes. The cytochrome *c* oxidase subunits (*cox1-3*), cytochrome *b* (*cob*), NADH dehydrogenase components (*nad1-6*), and rRNAs (*rrnL* and *rrnS*) are as indicated.

Our sequencing revealed the *S. retorta* mitogenome to be 15,652 bp in length (Fig. 1), consistent with reported mitogenomic lengths in other Lepidopteran species such as *Thitarodes pui* (Hepialidae; 15,064 bp) and *Plutella xylostella* (Plutellidae; 16,179 bp). We then aligned the *S. retorta* mitogenome sequences with those of other Lepidopteran species, enabling us to identify 13 PCGs (*atp6, atp8, cox1, cox2, cox3, cytb, nad1, nad2, nad3, nad4, nad5, nad6,* and *nad4L*), two rRNAs (large and small rRNA), 22 tRNAs, and a non-coding A + T-rich region that is conserved within the mitogenome of most known animal species^5^ (Table 1). The *S. retorta* mitogenome also harbored a *trnM*-*trnI*-*trnQ* gene arrangement that was distinct from the ancestral *trnI*-*trnQ*-*trnM* gene order^6^.

**Table 1:** List of annotated mitochondrial genes of *S. retorta.*

Gene	Direction	Location	Size	Anti codon	Start codon	Stop codon	Intergenic nucleotides
tRNA^Met^	F	1–68	68	CAT	–	–	0
tRNA^Ile^	F	75–141	69	GAT	–	–	6
tRNA^Gln^	R	139–207	69	TTG	–	–	− 3
nad2	F	267–1280	1014		ATT	TAA	59
tRNA^Trp^	F	1279–1346	68	TCA	–	–	− 2
tRNA^Cys^	R	1339–1406	68	GCA	–	–	− 8
tRNA^Tyr^	R	1420–1486	67	GTA	–	–	13
cox1	F	1494–3032	1539		ATG	TAA	7
tRNA^Leu(UUR)^	F	3028–3094	67	TAA	–	–	− 5
cox2	F	3095–3776	683		ATG	T	0
tRNA^Lys^	F	3777–3847	71	CTT	–	–	0
tRNA^Asp^	F	3847–3913	67	GTC	–	–	− 1
atp8	F	3923–4081	159		ATA	TAA	9
atp6	F	4075–4752	678		ATG	TAA	− 7
cox3	F	4801–5589	789		ATG	TAA	48
tRNA^Gly^	F	5592–5657	66	TCC	–	–	2
nad3	F	5658–6011	354		ATT	TAA	0
tRNA^Ala^	F	6014–6078	65	TGC	–	–	2
tRNA^Arg^	F	6086–6152	67	TCG	–	–	7
tRNA^Asn^	F	6162–6227	66	GTT	–	–	9
tRNA^Ser(AGN)^	F	6236–6301	66	GCT	–	–	8
tRNA^Glu^	F	6306–6372	67	TTC	–	–	4
tRNA^Phe^	R	6378–6445	68	GAA	–	–	5
nad5	R	6451–8163	1713		ATA	TAA	5
tRNA^His^	R	8194–8262	69	GTG	–	–	30
nad4	R	8266–9601	1335		ATG	T	3
nad4L	R	9665–9952	288		ATG	TAA	63
tRNA^Thr^	F	9970–10,034	65	TGT	–	–	17
tRNA^Pro^	R	10,035–10,099	65	TGG	–	–	0
nad6	F	10,137–10,640	504		ATT	TAA	37
cytb	F	10,692–11,843	1151		ATG	TAA	51
tRNA^Ser(UCN)^	F	11,855–11,921	67	TGA	–	–	11
nad1	R	11,968–12,906	939		ATG	TGA	47
tRNA^Leu(CUN)^	R	12,908–12,975	68	TAG	–	–	1
rrnL	R	12,976–14,388	1413	–	–	–	0
tRNA^Val^	R	14,389–14,455	67	TAC	–	–	0
rrnS	R	14,455–15,235	781	–	–	–	185
A + T-rich region		15,236–15,652	417				

The composition and skewness of the *S. retorta* mitogenome were compared to those of other Noctuoidea species (Table 2). The major strand was composed of A, T, G, and C nucleotides at relative frequencies of 35.59%, 41.23%, 7.37%, and 11.80%, respectively (80.83% A + T), thus exhibiting negative AT and GC skewness (− 0.020 and − 0.231, respectively). The AT skewness of other Noctuoidea mitogenomes has been found to range between 0.016 (*L. dispar*) and − 0.027 (*A. formosae*), whereas GC skewness values range from − 0.266 (*A. formosae*) to − 0.178 (*A. ipsilon*). The negative AT skewness in *S. retorta* indicates that there are more T residues than A residues, as previously reported for many Lepidopteran species including *A. formosae* (− 0.027), and *A. ipsilon* (− 0.006) (Table 2).

**Table 2:** The composition and skewness of mitogenomes of different *Noctuoidea* species*.*

Species	Size(bp)	A%	G%	T%	C%	A + T%	ATskewness	GCskewness
**Whole genome**								
*S. retorta*	15,652	39.59	7.37	41.23	11.80	80.83	− 0.020	− 0.231
*L. dispar*	15,569	40.58	7.57	39.30	12.55	79.88	0.016	− 0.248
*L. melli*	15,418	39.38	8.72	39.29	13.06	78.67	0.001	− 0.199
*H. cunea*	15,481	40.58	7.55	39.81	12.06	80.39	0.010	− 0.230
*A. formosae*	15,463	38.67	7.53	40.83	12.98	79.49	− 0.027	− 0.266
*A. ipsilon*	15,377	40.38	7.71	40.87	11.04	81.25	− 0.006	− 0.178
**PCG**								
*S. retorta*	11,134	33.73	10.83	45.27	10.17	79.00	− 0.146	0.031
*L. dispar*	11,227	39.67	8.44	38.16	13.73	77.83	0.019	− 0.239
*L. melli*	11,120	38.47	9.17	38.17	14.19	76.64	0.004	− 0.215
*H. cunea*	11,198	39.98	8.35	38.61	13.06	78.59	0.017	− 0.220
*A. formosae*	11,217	38.18	8.28	39.62	13.92	77.80	− 0.019	− 0.254
*A. ipsilon*	11,226	39.69	8.44	40.14	11.72	79.83	− 0.006	− 0.163
**tRNA**								
*S. retorta*	1478	41.95	10.89	39.45	7.71	81.39	0.031	0.171
*L. dispar*	1459	41.60	7.95	39.48	10.97	81.08	0.026	− 0.160
*L. melli*	1486	40.58	8.55	40.24	10.63	80.82	0.004	− 0.109
*H. cunea*	1463	41.83	7.86	39.99	10.32	81.82	0.022	− 0.135
*A. formosae*	1457	40.43	7.96	40.36	11.26	80.78	0.001	− 0.172
*A. ipsilon*	1465	41.23	8.12	40.48	10.17	81.71	0.009	− 0.112
**rRNA**								
*S. retorta*	2051	43.88	10.58	40.52	5.02	84.40	0.040	0.356
*L. dispar*	2150	42.79	4.79	41.81	10.60	84.60	0.012	− 0.377
*L. melli*	2233	42.23	4.93	41.96	10.88	84.19	0.003	− 0.376
*H. cunea*	2234	42.08	4.92	42.75	10.25	84.83	− 0.008	− 0.351
*A. formosae*	2163	38.93	4.72	44.85	11.51	83.77	− 0.071	− 0.418
*A. ipsilon*	2162	41.58	5.00	43.57	9.85	85.15	− 0.023	− 0.327
**A + T-rich region**								
*S. retorta*	417	45.08	1.68	48.68	4.56	93.76	− 0.038	− 0.462
*L. dispar*	435	40.58	7.57	39.30	12.55	79.88	0.016	− 0.248
*L. melli*	338	43.20	1.48	51.18	4.14	94.38	− 0.085	− 0.473
*H. cunea*	357	45.66	1.12	49.30	3.92	94.96	− 0.038	− 0.556
*A. formosae*	482	42.95	2.90	49.79	4.36	92.74	− 0.074	− 0.201
*A. ipsilon*	332	46.08	1.51	48.80	3.61	94.88	− 0.029	− 0.410

### PCGs and codon usage

We identified 13 total PCGs in the *S. retorta* mitogenome, spanning 71.1% of the mitogenome sequence (11,134 bp). These genes were between 159 bp (*atp8*) and 1713 bp (*nad5*) in length. This is consistent with findings from other Lepidopteran species in which these two mitogenes are often the shortest and longest, respectively^11,13^. All detected PCGs began with an ATN codon (2 ATA, 3 ATT, and 8 ATG). Atypical *cox1* start codons (including TTAG, ACG, and TTG) have been reported in the mitogenomes of a range of insect species^14–16^. The TAA stop codon was the most common among these PCGs (*nad2, cox1, atp8, atp6, cox3, nad3, nad5, nad4L, nad6, cytb*), while *nad1* utilized a TAG stop codon, and *nad4* and *cox2* harbored incomplete stop codons (T) (Table 1). This is a common conserved mitogenomic feature among invertebrates^17–19^, and this single T residue can be still be recognized by endonucleases during polycistronic pre-mRNA transcription, with polyadenylation from contiguous PCGs ultimately yielding a functional stop codon^5,20,21^.

**Figure 2:**
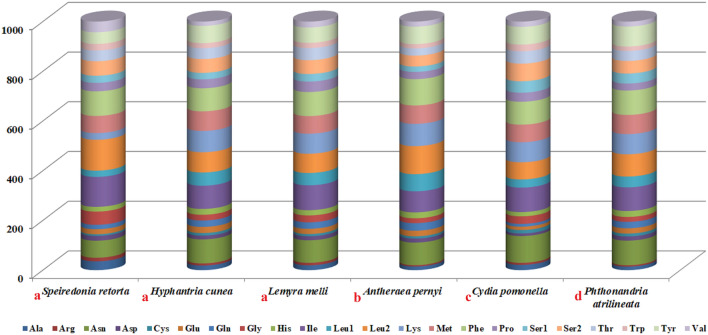
A comparative assessment of mitogenomic codon usage among Lepidopteran insects. Superfamily membership is indicated above species names using lowercase letters (a: Noctuoidea, b: Bombycoidea, c: Tortricoidea, d: Geometroidea).

**Figure 3:**
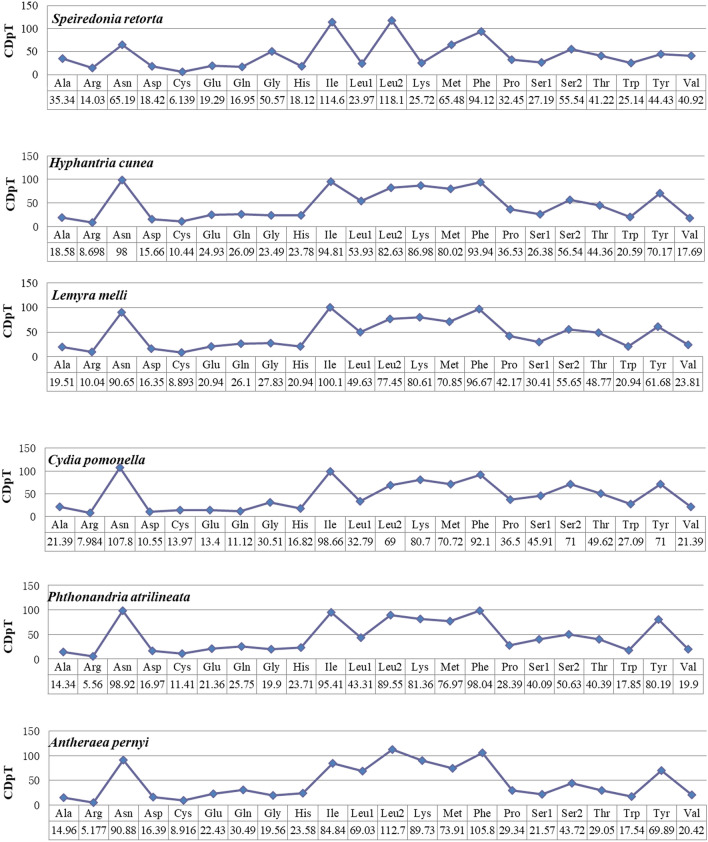
The distribution of codons among Lepidopteran species. *CDspT* codons per thousand codons.

Next, we assessed codon usage among a range of Lepidopteran species, including three Noctuoidae members as well as Bombycoidea, Tortricoidea, and Geometroidea members (Fig. 2). Through this approach, we found Asn, Ile, Leu2, Lys, Met, Phe, and Tyr to be the amino acids that were used most often. In these 6 species, we identified 4 codon families with at least 80 codons per thousand codons (Ile and Phe), and 3 with a minimum of 60 codons per thousand codons (Leu2, Met and Asn). The Arg and Cys codon families were the least represented. The codon distributions were consistent among the three Noctuoidae species analyzed herein, with the exception of Lys, which was rarely encoded in *S. retorta* (Fig. 3).

**Figure 4:**
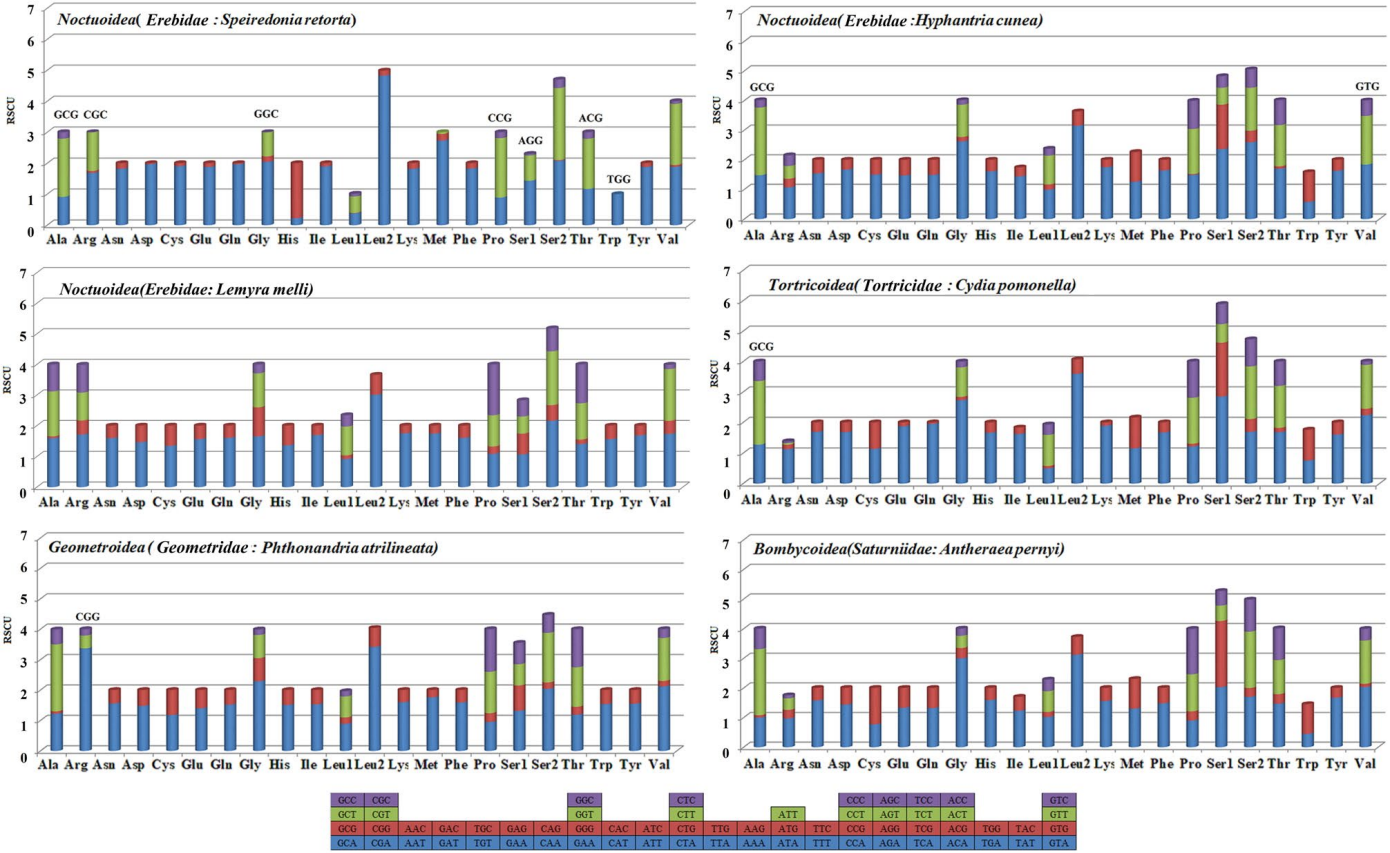
Mitogenome Relative Synonymous Codon Usage (RSCU) for the four Lepidopteran superfamilies, with codon families on the *x*-axis. Any codons above the bar were absent within the mitogenome.

The RCSU was next assessed in the PCGs encoded by the mitogenomes of the four Lepidopteran superfamilies (Fig. 4). We found that the PCGs of *S. retorta* contained all possible combinations of codons other than GCG, CGC, GGC, AGG, CCG, ACG, and TGG. This lack of GC-rich codons is common to many other Lepidopterans including *H. cunea* (GCG and GTG), *P. atrilineata* (CGG), and *C. pomonella* (GCG). Utilized codons showed a bias towards A + T content, contributing to the overall mitogenomic AT-bias that was observed both in *S. retorta* and across other insect mitogenomes^19,22^.

### Ribosomal and transfer RNA genes

Two rRNA genes were identified within the *S. retorta* mitogenome, consistent with findings in most other animal species. The 1413 bp large ribosomal RNA gene (*rrnL*) was located in between tRNA Leu (CUN) and tRNA Val, while the 781 bp small ribosomal RNA gene (*rrnS*) was between tRNA Val and the A + T-rich region (Table 1). These rRNA genes were A + T rich (84.40%), falling within the range observed for other Noctuoidae species including *A. formosae* (83.77%) and *A. ipsilon* (85.15%). These rRNA AT and GC skewness values have been found to be negative in the majority of analyzed Lepidopteran mitogenomes^17^, however, in *S. retorta* these values were positive (0.040 and 0.356, respectively), as has been reported in a subset of prior studies^23,24^.

**Figure 5:**
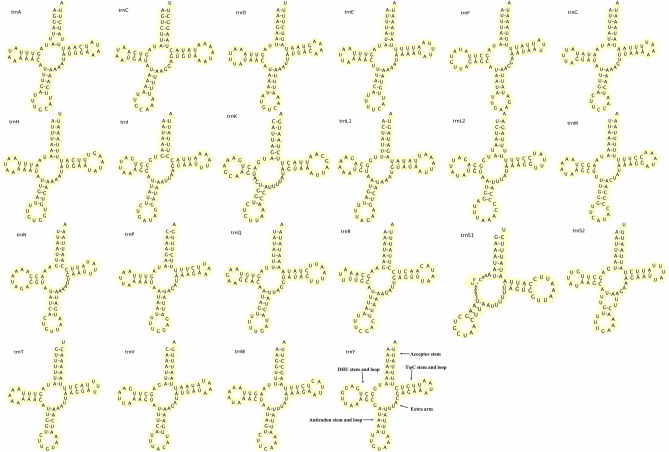
Predicted secondary structures for the 22 *S. retorta* mitogenome-encoded tRNAs.

We identified a full set of 22 tRNA genes (65–71 nucleotides long) in the *S. retorta* mitogenome, consistent with findings from other Lepidopterans. These tRNA regions were heavily A + T biased (81.39%), with positive AT and GC skewness (0.031 and 0.171, respectively; Table 2). All of these tRNAs exhibited expected cloverleaf-like secondary structures, although the DHU stem was lacking from *trnS1* (Fig. 5), as has previously been observed in other Lepidopterans^23^. Furthermore, 8/22 tRNAs were encoded on the L-strand while 12/22 were encoded on the H-strand.

### Overlapping and intergenic spacer regions

**Figure 6:**
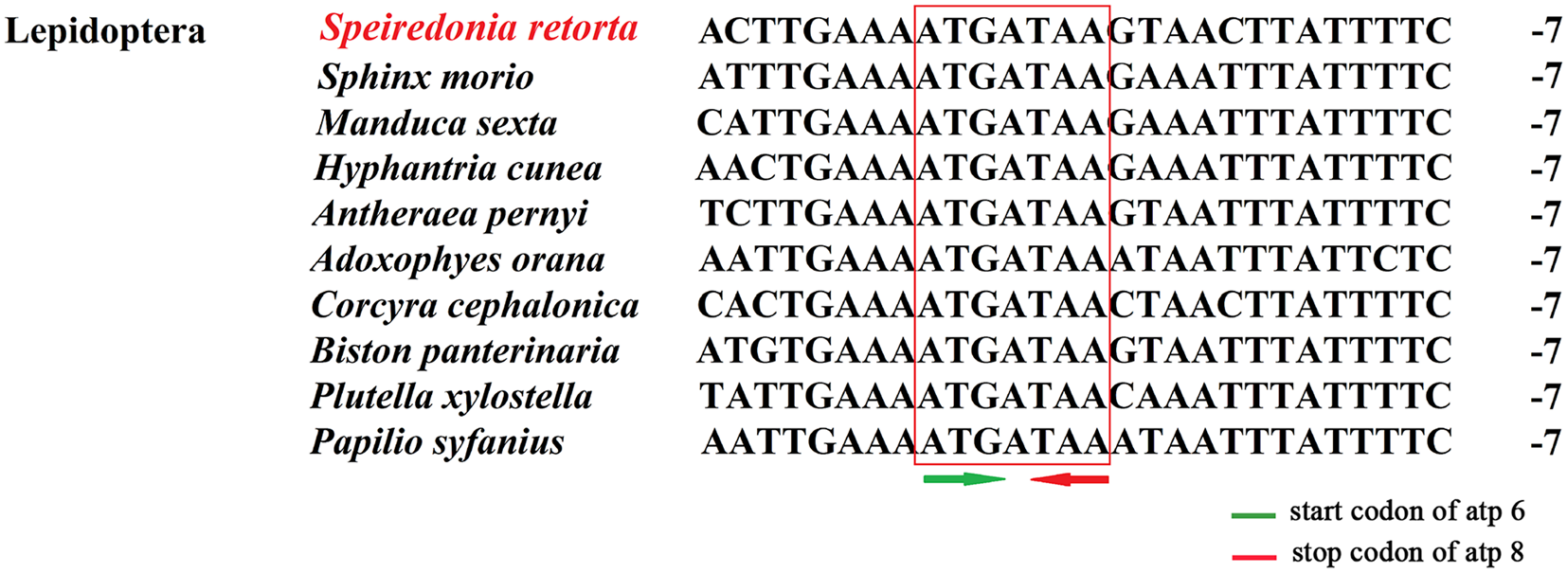
Alignment of *atp8* and *atp6* overlap among Lepidopteran species and other insects, with the number of intergenic nucleotides shown on the right.

**Figure 7:**
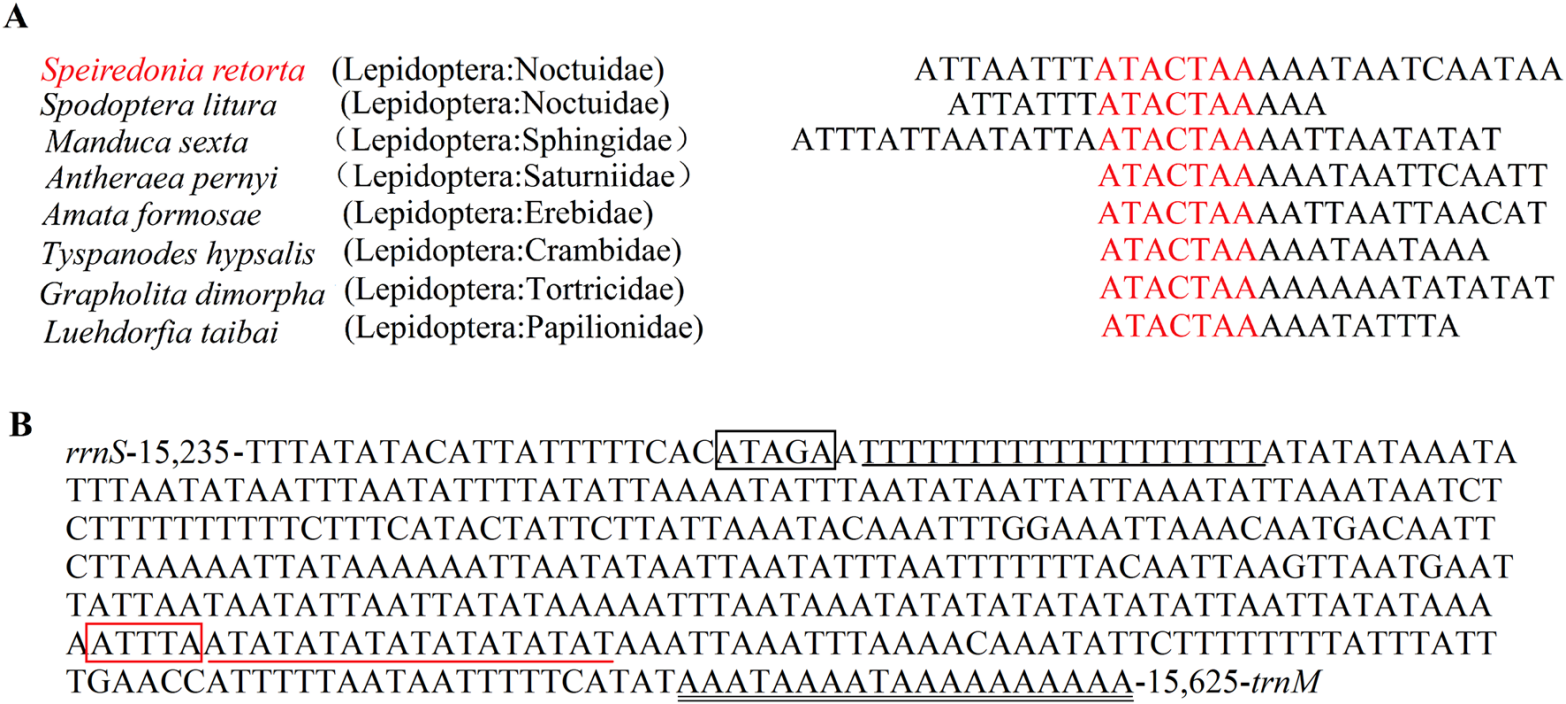
(**A**) The intergenic spacer region between *trnS2* (UCN) and *nad1* was aligned for multiple Lepidopteran species, with the conserved ‘ATACTAA’ motif being highlighted. (**B**) Features of the *S. retorta* A + T-rich region are shown in the reverse strand, with the ATATGA motif being highlighted. Double underlines were used to mark the poly-A region, while a single underline was used to mark the poly-T region. A dotted underline was used to mark single microsatellite T/A repeats.

There are 6 overlapping regions in the *S. retorta* mitogenome. These regions range from 1 to 8 bp in size (26 bp total), with the longest region of overlap being located between *trnC* and *trnY* (Table 1). When we aligned the region of overlap between *atp6* and *atp8,* we found that *S. retorta* mitogenome contained an ATGATAA nucleotide sequence common to other Lepidopterans (Fig. 6). In addition, we identified the ‘ATACTAA’ motif within the 17 bp region between *trnS2* (UCN) and *nad1* (Fig. 7A), with this motif being highly conserved in insect mitogenomes in addition to being a potential mitochondrial transcription termination peptide-binding site (mtTERM protein)^25^.

### A + T-rich region analysis

An A + T-rich region that was 417 bp long was detected between *rrnS* and *trnM* in the *S. retorta* mitogenome. This region was composed of 93.76% AT residues and exhibited negative AT and GC skewness (− 0.038 and − 0.462, respectively) (Table 2). This region contained a number of short repeated sequences, such as a 19 bp poly-T region that flanked an ‘ATAGA’ motif near *rrnS*, an ‘ATTTA’ roughly in the center of this A + T-rich region, as well as a poly-A element that was located upstream relative to *trnM*, consistent with mitogenomic findings from other Lepidopteran species (Fig. 7B). In addition, while the exact poly-T region length varies among Lepidopterans^4,17,26^, the ATAGA motif is highly conserved^27^.

### Phylogenetic analyses

**Figure 8:**
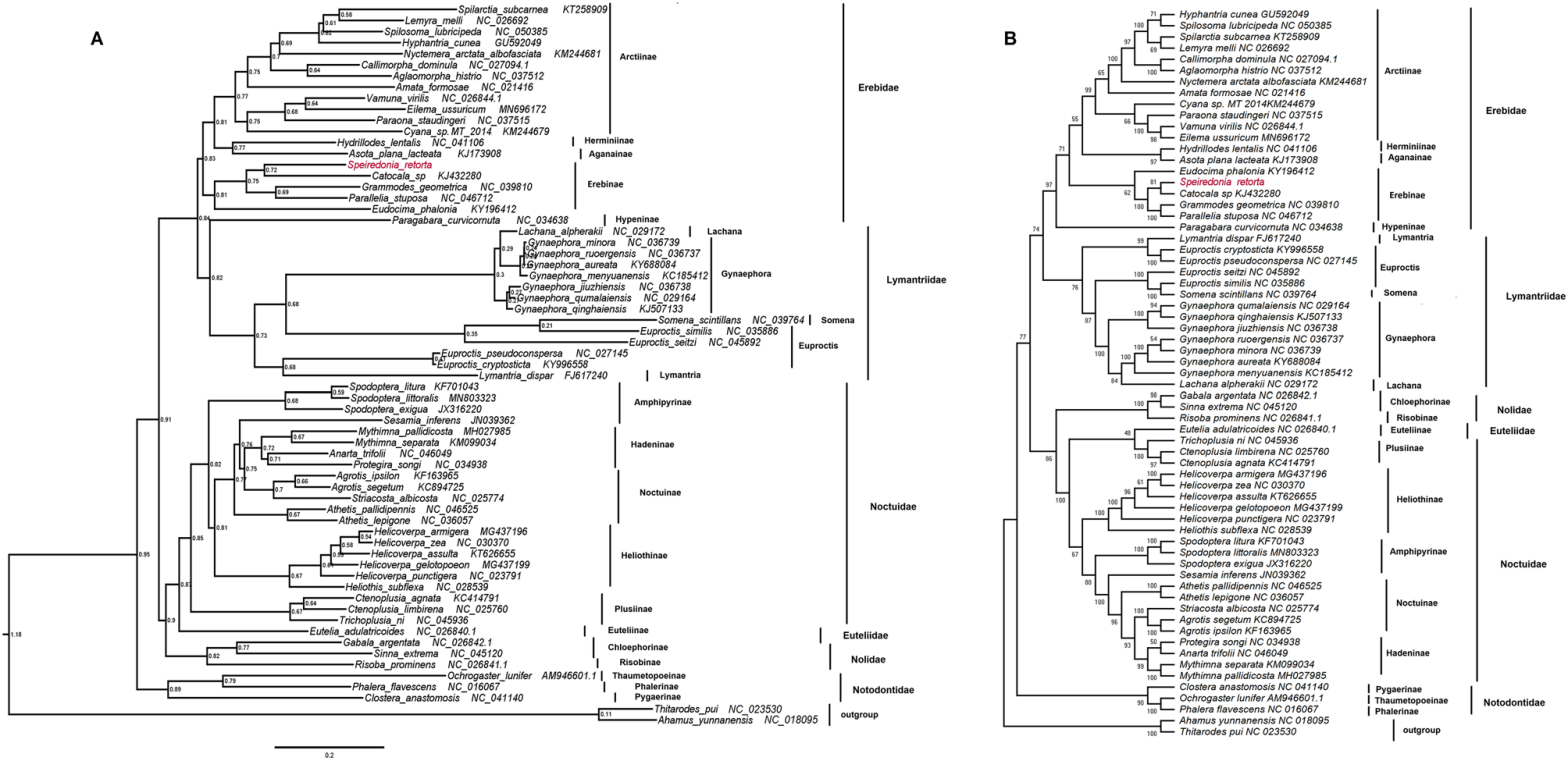
Tree showing the phylogenetic relationships among Noctuoidea insects, constructed using (**A**) Bayesian inference (BI). (**B**) Maximum Likelihood method (ML). As outgroups for this analysis, we utilized *Ahamus yunnanensis* (NC_018095) and *Thitarodes pui* (NC_023530).

A number of recent studies have explored phylogenetic relationships among Noctuoidea species. In one recent analysis, Zahiri et al. proposed the following relationship among these families: (Notodontidae + (Euteliidae + (Noctuidae + (Erebidae + Nolidae))))^28^. In contrast, Yang et al. published another study in which the following grouping scheme was proposed: (Notodontidae + (Erebidae + (Nolidae + (Euteliidae + Noctuidae))))^29^. In this study, we utilized BI and ML methods as well as the MAFFT alignment approach in order to explore the relationship between *S. retorta* and other Noctuidae insects according to its mitogenome sequence. We utilized the NT dataset to conduct phylogenetic analyses of 65 full mitogenomes which were representative of six Noctuoidea families (Erebidae, Lymantriidae, Euteliidae, Noctuidae, Notodontidae, and Nolidae). For outgrouping purposes, we additionally utilized the mitogenomes of *Ahamus yunnanensis* (NC_018095) and *Thitarodes pui* (NC_023530) in the present analysis (Fig. 8). According to Homziak’s study, our analyses revealed a topology within Erebinae that was as follows: ((*Catocala *sp + *Speiredonia retorta*) + (*Grammodes geometrica* + *Parallelia stuposa*) + Eudocima phalonia). The result indicated that Catocalini belongs to Erebinae subfamily^30^, as confirmed via BI (Fig. 8A) and ML (Fig. 8B) analyses. This approach further confirmed that *S. retorta* is a member of Erebidae: (Notodontidae + (Erebidae + Lymantriidae + (Nolidae + (Euteliidae + Noctuidae)))). This is distinct from a study conducted by Zahiri et al. that yielded different phylogenetic results: (Notodontidae + (Euteliidae + (Noctuidae + (Erebidae + Nolidae))))^28^.

*S. retorta* has previously been identified as a member of the Noctuoidea superfamily within the Erebidae family and the Erebinae subfamily^30^. Our data were consistent with this hypothesis. Even so, our findings were distinct from those of the prior study with respect to some of the identified relationships, suggesting that the sequencing of the mitogenomes of additional Noctuoidea species will be required to more accurately resolve these phylogenetic relationships.

## Materials and methods

### Insects and DNA collection

Samples of *S. retorta* were obtained from Bengbu Medical College, Anhui Province, China. The collected specimens were identified as *Speiredonia retorta* based on their morphological characteristics by a taxonomist of the Department of Entomology, Anhui Agricultural University, Hefei, China (AHAU). After a careful examination of the morphological characteristics and the comparison of voucher specimens to the referenced publications regarding Healthy plantations from the Forest Science Institute of Vietnam^1^. A Genomic DNA Extraction Ki (Aidlab Co., Beijing, China) was used to extract DNA from these samples, after which 1% agarose gel electrophoresis (AGE) was used to evaluate DNA quality. Samples were then used for mitogenome isolation.

### Mitogenome sequencing

The *S. retorta* mitogenome was amplified using 12 primer pairs designed based on known conserved mitogenome sequences in other Lepidopteran species (Table 3; BGI Group Co., Guangdong, China)^2,31,32^. An Eppendorf Mastercycler and Mastercycler gradient were used to amplify mitogenomic sequences in a 50 μL reaction volume containing 35 μL dH_2_O, 5 μL 10 × Taq buffer (Mg^2+^ plus), 4 μL dNTP (25 mM), 1.5 μL DNA, 2 μL of each primer (F + R; 10 μM) as well as 0.5 μL TaqDNA polymerase (Takara Co., Dalian, China). Thermocycler settings were: 94 °C for 4 min; 38 cycles of 94 °C for 30 s, 48–59 °C for 1–3 min (based on predicted fragment length); 72 °C for 10 min. Samples were then separated via 1% AGE and collected with a DNA gel extraction kit (Transgen Co., Beijing, China), after which direct sequencing was conducted with appropriate PCR primers.

**Table 3:** Details of the Lepidopteran mitogenomes used in this study.

Superfamily	Family	Subfamily	Species	GenBank accession no
Noctuoidea	Erebidae	Erebinae	*Speiredonia retorta*	MT013356
Noctuoidea	Erebidae	Erebinae	*Catocala *sp.	KJ432280
Noctuoidea	Erebidae	Erebinae	*Eudocima phalonia*	KY196412
Noctuoidea	Erebidae	Erebinae	*Grammodes geometrica*	NC_039810
Noctuoidea	Erebidae	Erebinae	*Parallelia stuposa*	NC_046712
Noctuoidea	Erebidae	Aganainae	*Asota plana lacteata*	KJ173908
Noctuoidea	Erebidae	Arctiinae	*Amata formosae*	NC_021416
Noctuoidea	Erebidae	Arctiinae	*Aglaomorpha histrio*	NC_037512
Noctuoidea	Erebidae	Arctiinae	*Callimorpha dominula*	NC_027094.1
Noctuoidea	Erebidae	Arctiinae	*Cyana *sp.* MT-2014*	KM244679
Noctuoidea	Erebidae	Arctiinae	*Eilema ussuricum*	MN696172
Noctuoidea	Erebidae	Arctiinae	*Hyphantria cunea*	GU592049
Noctuoidea	Erebidae	Arctiinae	*Lemyra melli*	NC_026692
Noctuoidea	Erebidae	Arctiinae	*Nyctemera arctata albofasciata*	KM244681
Noctuoidea	Erebidae	Arctiinae	*Paraona staudingeri*	NC_037515
Noctuoidea	Erebidae	Arctiinae	*Spilarctia subcarnea*	KT258909
Noctuoidea	Erebidae	Arctiinae	*Spilosoma lubricipeda*	NC_050385
Noctuoidea	Erebidae	Arctiinae	*Vamuna virilis*	NC_026844.1
Noctuoidea	Erebidae	Herminiinae	*Hydrillodes lentalis*	NC_041106
Noctuoidea	Erebidae	Hypeninae	*Paragabara curvicornuta*	NC_034638
Noctuoidea	Euteliidae	Euteliinae	*Eutelia adulatricoides*	NC_026840.1
Noctuoidea	Lymantriidae	Lachana	*Lachana alpherakii*	NC_029172
Noctuoidea	Lymantriidae	Euproctis	*Euproctis pseudoconspersa*	NC_027145
Noctuoidea	Lymantriidae	Euproctis	*Euproctis cryptosticta*	KY996558
Noctuoidea	Lymantriidae	Euproctis	*Euproctis seitzi*	NC_045892
Noctuoidea	Lymantriidae	Euproctis	*Euproctis similis*	NC_035886
Noctuoidea	Lymantriidae	Gynaephora	*Gynaephora aureata*	KY688084
Noctuoidea	Lymantriidae	Gynaephora	*Gynaephora jiuzhiensis*	NC_036738
Noctuoidea	Lymantriidae	Gynaephora	*Gynaephora menyuanensis*	KC185412
Noctuoidea	Lymantriidae	Gynaephora	*Gynaephora minora*	NC_036739
Noctuoidea	Lymantriidae	Gynaephora	*Gynaephora qinghaiensis*	KJ507133
Noctuoidea	Lymantriidae	Gynaephora	*Gynaephora qumalaiensis*	NC_029164
Noctuoidea	Lymantriidae	Gynaephora	*Gynaephora ruoergensis*	NC_036737
Noctuoidea	Lymantriidae	Lymantria	*Lymantria dispar*	FJ617240
Noctuoidea	Lymantriidae	Somena	*Somena scintillans*	NC_039764
Noctuoidea	Noctuidae	Amphipyrinae	*Sesamia inferens*	JN039362
Noctuoidea	Noctuidae	Amphipyrinae	*Spodoptera exigua*	JX316220
Noctuoidea	Noctuidae	Amphipyrinae	*Spodoptera littoralis*	MN803323
Noctuoidea	Noctuidae	Amphipyrinae	*Spodoptera litura*	KF701043
Noctuoidea	Noctuidae	Hadeninae	*Anarta trifolii*	NC_046049
Noctuoidea	Noctuidae	Hadeninae	*Mythimna separata*	KM099034
Noctuoidea	Noctuidae	Hadeninae	*Mythimna pallidicosta*	MH027985
Noctuoidea	Noctuidae	Hadeninae	*Protegira songi*	NC_034938
Noctuoidea	Noctuidae	Heliothinae	*Helicoverpa armigera*	MG437196
Noctuoidea	Noctuidae	Heliothinae	*Helicoverpa gelotopoeon*	MG437199
Noctuoidea	Noctuidae	Heliothinae	*Helicoverpa punctigera*	NC_023791
Noctuoidea	Noctuidae	Heliothinae	*Helicoverpa zea*	NC_030370
Noctuoidea	Noctuidae	Heliothinae	*Helicoverpa assulta*	KT626655
Noctuoidea	Noctuidae	Heliothinae	*Heliothis subflexa*	NC_028539
Noctuoidea	Noctuidae	Noctuinae	*Athetis lepigone*	NC_036057
Noctuoidea	Noctuidae	Noctuinae	*Athetis pallidipennis*	NC_046525
Noctuoidea	Noctuidae	Noctuinae	*Agrotis ipsilon*	KF163965
Noctuoidea	Noctuidae	Noctuinae	*Agrotis segetum*	KC894725
Noctuoidea	Noctuidae	Noctuinae	*Striacosta albicosta*	NC_025774
Noctuoidea	Noctuidae	Plusiinae	*Ctenoplusia agnata*	KC414791
Noctuoidea	Noctuidae	Plusiinae	*Ctenoplusia limbirena*	NC_025760
Noctuoidea	Noctuidae	Plusiinae	*Trichoplusia ni*	NC_045936
Noctuoidea	Nolidae	Chloephorinae	*Gabala argentata*	NC_026842.1
Noctuoidea	Nolidae	Chloephorinae	*Sinna extrema*	NC_045120
Noctuoidea	Nolidae	Risobinae	*Risoba prominens*	NC_026841.1
Noctuoidea	Notodontidae	Phalerinae	*Phalera flavescens*	NC_016067
Noctuoidea	Notodontidae	Pygaerinae	*Clostera anastomosis*	NC_041140
Noctuoidea	Notodontidae	Thaumetopoeinae	*Ochrogaster lunifer*	AM946601.1
Hepialoidea	Hepialidae	Ahamus	*Ahamus yunnanensis*	NC_018095
Hepialoidea	Hepialidae	Thitarodes	*Thitarodes pui*	NC_023530

### Sequence assembly and annotation

NCBI BLAST (http://blast.ncbi.nlm.nih.gov/Blast) and SeqMan II (DNASTAR Inc.; WI, USA) were used to annotate the sequenced mitogenome. The Invertebrate Mitochondrial Genetic Code was used to identify putative proteins encoded by specific PCGs. An approach previously detailed by Junqueira et al.^33^ was used to assess skewness, with base composition being assessed as follows: AT skew = [A − T]/[A + T], GC skew = [G − C]/[G + C].

The tRNAscan-SE program (http://lowelab.ucsc.edu/tRNAscan-SE/) was used to identify tRNA genes^34^, with additional predictions being made based upon sequences capable of adopting a tRNA secondary structure and containing an anticodon. Tandem repeats within the A + T-rich region were identified with a tandem repeat finder application (http://tandem.bu.edu/trf/trf.html)^35^.

### Codon usage and RCSU

We assessed codon usage across a range of Lepidopteran species, including three Noctuoidae members as well as Bombycoidea and Geometroidea members that were closely related to Noctuoidae species and Tortricoidea species that were far more distantly related to these species^24,36^ (Fig. 2). MEGA7.0 was used to calculate relative synonymous codon usage (RSCU) values^37^.

### Phylogenetic analysis

Lepidopteran phylogenetic relationships were evaluated by utilizing all 64 Noctuoidea mitogenomes available from Genbank (Table 3), with the *Ahamus yunnanensis* (NC_018095) and *Thitarodes pui* (NC_023530) mitogenomes serving as an outgroup for this analysis. MAFFT was then used to conduct multiple alignments of concatenated nucleotide sequences for the 13 PCGs using default settings^38^, and the resultant concatenated sequences were used for phylogenetic analyses, which were conducted via the Maximum Likelihood (ML) method using MEGA7.0^37^ and via a Bayesian Inference (BI) approach using MrBayes v3.2^39^. A total of 1000 bootstrap replicates were used for ML analysis in order to develop phylogenetic trees. For the BI analysis, the GTR + I + G model was used for the analyzed nucleotide sequences, with MrModeltest 2.3 being used in accordance with Akaike's information criterion (AIC). In the BI analysis, four simultaneous MCMC chains were run for 10,000,000 generations, with sampling being conducted in 1000 generation intervals with a 2500 generation burn-in. FigTree v1.4.2 (http://tree.bio.ed.ac.uk/software/figtree/) was then used to visualize the resultant phylogenetic trees.
